# Effects of Latin dance exercise on body image and social anxiety in female college students

**DOI:** 10.3389/fpsyg.2026.1958896

**Published:** 2026-10-09

**Authors:** Luyao Wang, Zhenzhen Li, Yiming Liu, Xutao Lyu

**Affiliations:** 1 School of Physical Education, Henan University, Kaifeng, China; 2 School of Physical Education, Henan Kaifeng College of Science Technology and Communication, Kaifeng, China

**Keywords:** body image, female college students, indirect effect, Latin dance, social anxiety

## Abstract

**Background:**

Latin dance combines bodily expression with social interaction and may influence body image and social anxiety, but randomized controlled evidence remains limited. This study evaluated a 10-week Latin dance exercise intervention and examined an exploratory statistical indirect effect involving an aggregate body image index among female college students.

**Methods:**

Of 103 students recruited, 88 eligible participants were randomly allocated 1:1 to the Latin dance or usual-activity control group (*n* = 44 each). Body image and social anxiety were assessed at baseline and immediately after the intervention using five appearance-related subscales of the Chinese Multidimensional Body-Self Relations Questionnaire and the Social Avoidance and Distress Scale, respectively. Primary analyses of covariance evaluated post-intervention group differences after adjustment for the corresponding baseline scores. Secondary mixed-design analyses of variance examined Group × Time differences, and a baseline-adjusted PROCESS Model 4 with 5,000 bootstrap resamples estimated the exploratory statistical indirect effect.

**Results:**

Primary baseline-adjusted analyses showed that, compared with the control group, the Latin dance group had lower adjusted post-intervention scores for Overweight Preoccupation, Self-Classified Weight, Social Avoidance, Social Distress, and the Social Avoidance and Distress overall mean score, and higher adjusted scores for Appearance Evaluation, Body Areas Satisfaction, and the aggregate body image index (all *p* < 0.001, η_*p*_^2^ = 0.218–0.606). Appearance Orientation did not differ between groups (*p* = 0.700, η_*p*_^2^ = 0.002). Secondary Group × Time analyses showed differential changes in Overweight Preoccupation, Self-Classified Weight, Appearance Evaluation, Body Areas Satisfaction, and all three social anxiety measures, but not in Appearance Orientation or the aggregate body image index. The exploratory model yielded a statistically significant indirect effect involving the aggregate body image index (−0.43, bootstrap 95% CI [−0.61, −0.26]), whereas the direct effect was not statistically significant (−0.08, 95% CI [−0.30, 0.13]).

**Conclusion:**

Assignment to the Latin dance intervention was associated with lower adjusted post-intervention Overweight Preoccupation and social anxiety scores and higher Appearance Evaluation and Body Areas Satisfaction scores than usual activity. The exploratory indirect effect requires confirmation in larger, rigorously designed studies.

## 1 Introduction

Since Schilder's original 1935 formulation, body image research has broadened from an emphasis on negative experiences, such as body dissatisfaction and body surveillance, to include body acceptance and other positive body-related experiences (Schilder, 2013; Grogan, 2016; Yang et al., 2023). Accordingly, the present study conceptualizes body image as a multidimensional construct encompassing both positive and negative body-related experiences and evaluations. As an important component of self-evaluation, body image is associated with both intrapersonal experience and interpersonal functioning. It may shape how individuals perceive themselves in the presence of others and may therefore be related to social anxiety.

Early clinical discussions of social anxiety often centered on social phobia, particularly the persistent fear of scrutiny in social or performance situations (Pélissolo and Lépine, 1995; Marks and Gelder, 1966). In the present study, social anxiety refers to tension, unease, and other negative emotional experiences arising in actual or anticipated social situations because of concerns about negative evaluation, humiliation, or embarrassment. It may also involve heightened self-focused attention and avoidance or distress in social situations (Watson and Friend, 1969; Rapee and Heimberg, 1997). Among the forms of negative evaluation that individuals may fear, judgments concerning appearance and the body are particularly salient (Lundgren et al., 2004; Hart et al., 2008). Expectations about how others will evaluate one's appearance may be shaped by one's own body image. Body image and social anxiety may therefore be closely related.

Objectification theory provides one framework for understanding this relationship. Repeated exposure to sociocultural environments that emphasize women's appearance may encourage women to internalize an observer's perspective as a standard for self-evaluation, thereby fostering self-objectification and habitual body surveillance. Persistent monitoring of how the body appears to others may increase negative body-related thoughts and feelings and contribute to body dissatisfaction. In social situations, such monitoring may also heighten concerns about appearance-based evaluation and increase anxiety or avoidance (Fredrickson and Roberts, 1997). Consistent with this account, negative body image has been associated with greater evaluative anxiety in interpersonal contexts (Cash et al., 2004).

Female college students constitute a relevant population in which to examine these processes. In a cross-sectional study of Omani university students, body image dissatisfaction was more prevalent among women than men (Al Riyami et al., 2024). Romantic and dating contexts also become increasingly salient during emerging adulthood (Arnett, 2000), and body image and social physique anxiety have been associated with dating anxiety in this population (Swami et al., 2021). In addition, frequent exposure to idealized images on social media may encourage upward appearance comparison and the internalization of sociocultural appearance standards (Tiggemann et al., 2018; Lee and Lee, 2021; Fioravanti et al., 2022; Jung et al., 2022). These processes may undermine body satisfaction and heighten sensitivity to others' evaluations. Negative physical self-perceptions have, in turn, been associated with greater social anxiety among college students (Wan et al., 2024). These social and developmental contexts support the relevance of examining body image and social anxiety among female college students.

Evidence from exercise interventions suggests that physical activity may benefit both anxiety and body image, although its effects vary across outcomes and exercise modalities. Meta-analytic evidence indicates that physical activity interventions can reduce anxiety symptoms among college students, but the certainty and consistency of the evidence remain limited (Lin and Gao, 2023; Huang et al., 2024). Exercise interventions have also produced improvements in body image (Hausenblas and Fallon, 2006; Campbell and Hausenblas, 2009). These changes may be more closely related to perceived physical changes and physical self-perceptions than to objective physical changes (Martin Ginis et al., 2012; Salci and Martin Ginis, 2017). Nevertheless, evidence from studies specifically examining social anxiety remains limited, and randomized findings have not been conclusive (Zika and Becker, 2021).

Dance-specific evidence is more limited but is directly relevant to the present study. A 12-week randomized controlled trial found that Latin dance improved physical self-perceptions among college students, including body attractiveness and sport competence, with particularly evident changes among female participants (Hu et al., 2025). Other intervention studies reported reductions in social anxiety among left-behind children and in social physique anxiety among middle school girls following dance or Latin dance programs (Li et al., 2025; Liu et al., 2025). An earlier 12-week Latin dance exercise program also reported lower social physique anxiety among university students (Adilogullari, 2014), whereas an exercise intervention involving male high school students reported concurrent improvements in body image and social anxiety (Bolboli et al., 2013). However, these studies differed substantially in their populations, outcome measures, intervention formats, and research designs. Evidence specific to multidimensional body image and social anxiety among female college students therefore remains preliminary.

Latin dance may be particularly relevant because it combines bodily performance, rhythmic coordination, aesthetic expression, and social interaction. By requiring participants to perceive and adjust their posture and movements, Latin dance may shift attention from appearance-based evaluation toward bodily function and movement competence. This functional shift in attention may counter the observer-based self-monitoring described in objectification theory, thereby lessening habitual body surveillance and promoting more favorable body-related evaluations. Reduced appearance-based monitoring may, in turn, decrease concerns about negative evaluation and make expressive behavior in social situations less threatening. The group-based structure of Latin dance may additionally provide opportunities for social support and interpersonal connection. Group exercise participation has been associated with several forms of social support, and synchronized dance movements have been linked to stronger social bonding and interpersonal closeness (Golaszewski et al., 2022; Tarr et al., 2015, 2016). Dancesport participation has also been associated with psychological wellbeing, with perceived social support identified as a statistical mediator of this association (Chen et al., 2025).

Observational studies among college students have reported statistical indirect associations involving body image in the relationship between physical activity and social anxiety (Gan and Jiang, 2024; Huang and Jiang, 2024). Together, these findings provide a rationale for examining body image as a possible statistical mediator of the relationship between Latin dance exercise and social anxiety. However, the proposed processes should be interpreted cautiously. Latin dance also involves aesthetic presentation and public performance, which may heighten appearance awareness or evaluative concerns for some participants rather than consistently reducing them. Moreover, the present study did not directly measure self-objectification or body surveillance. These constructs therefore represent theoretically plausible processes and specific directions for future research rather than mechanisms directly tested in the present study.

Latin dance has been incorporated into university physical education courses and extracurricular fitness activities in China (Tang, 2010), supporting the practical feasibility of examining it as a university-based intervention. Nevertheless, previous research has three important limitations. First, studies have frequently examined general anxiety, body esteem, or social physique anxiety rather than simultaneously assessing multidimensional body image and social anxiety. Second, dance intervention studies have used heterogeneous populations and designs, and randomized evidence focusing specifically on female college students remains limited. Third, although observational studies have identified statistical associations among physical activity, body image, and social anxiety, the statistical indirect pathway involving body image has received limited examination within a randomized Latin dance intervention.

To address these limitations, the present study used a two-group randomized controlled design to evaluate a 10-week Latin dance exercise intervention among female college students. Participants were randomly assigned to either the Latin dance group or a usual-activity control group that received no structured intervention from the research team. Body image and social anxiety were assessed at baseline and immediately after the intervention. Baseline-adjusted ANCOVAs were used as the primary analyses of post-intervention group differences, whereas Group × Time interaction tests were used as secondary analyses of differential change.

Body image and social anxiety were treated as two main outcome domains. The body image outcomes comprised Appearance Evaluation, Appearance Orientation, Body Areas Satisfaction, Overweight Preoccupation, and Self-Classified Weight. Higher Appearance Evaluation and Body Areas Satisfaction scores and lower Overweight Preoccupation scores represented the expected favorable changes. Appearance Orientation and Self-Classified Weight were assessed as distinct dimensions of appearance investment and perceived weight classification, respectively, without treating either direction of change as uniformly favorable. The social anxiety outcomes comprised Social Avoidance, Social Distress, and the Social Avoidance and Distress overall mean score, with lower scores indicating less social anxiety. An aggregate body image index was additionally used in the statistical indirect-effect analysis rather than being designated as a single primary endpoint.

Based on the preceding theoretical arguments and empirical evidence, the present study proposed the following hypotheses. After adjustment for the corresponding baseline scores, participants assigned to the Latin dance group would have higher post-intervention Appearance Evaluation and Body Areas Satisfaction scores and lower Overweight Preoccupation scores than participants assigned to the control group (H1). Appearance Orientation and Self-Classified Weight would be interpreted separately because neither dimension has a uniformly favorable direction. For social anxiety, the Latin dance group would have lower adjusted post-intervention Social Avoidance, Social Distress, and Social Avoidance and Distress overall mean scores than the control group (H2). Finally, an exploratory baseline-adjusted statistical indirect effect of group assignment on the post-intervention Social Avoidance and Distress overall mean score involving the post-intervention aggregate body image index was expected (H3).

## 2 Materials and methods

### 2.1 Trial registration

At the time of study planning, this university-based randomized exercise intervention was not identified as falling within the scope of prospective trial-registration requirements. Consequently, the study was not prospectively registered before participant enrollment, and no trial registration identifier is available.

### 2.2 Participants

An a priori power analysis was conducted using G^*^Power 3.1, with “ANOVA: repeated measures, within-between interaction” selected as the statistical test. The analysis assumed two groups, two measurement occasions, and a non-sphericity correction of ε = 1.00. The remaining parameters were Cohen's *f* = 0.25, α = 0.05, power (1 – β) = 0.80, and a baseline-to-post-intervention correlation of 0.50. The required total sample size was 34 participants. After adjusting for an anticipated attrition rate of 20% and ensuring equal 1:1 allocation, the minimum target sample size was set at 44 participants.

Following institutional authorization, female college students enrolled at Henan Kaifeng College of Science Technology and Communication were recruited on campus. All participants provided informed consent before enrollment in the study. Eligible participants were currently enrolled female college students who were willing to complete the study procedures and had no contraindications to exercise, including cardiovascular or metabolic diseases. Students were excluded if they were concurrently participating in another structured exercise or psychological intervention or had a background in specialized athletic training. No eligibility thresholds were applied to the MBSRQ or SAD because the study targeted a general, non-clinical student population rather than students selected for elevated body-image concerns or social-anxiety symptoms. Of the 103 students who volunteered, 88 met the eligibility criteria and completed the baseline assessment before randomization. The final sample size resulted from recruitment and eligibility screening and exceeded the prespecified minimum.

After enrollment and baseline assessment, random values were generated using the RAND function in Microsoft Excel. Participants were ranked according to the generated values and allocated in a 1:1 ratio to the Latin dance or control group. The original computer-generated allocation list was retained. No random-number seed was recorded; therefore, the random sequence cannot be independently regenerated. No formal allocation-concealment mechanism was used. The Latin dance intervention group is hereafter referred to as the intervention group. χ^2^ tests revealed no statistically significant between-group differences in grade, place of origin, or only-child status, all *p* > 0.05 (Table 1). These results provided no evidence of baseline imbalance in these demographic characteristics. Participant flow through recruitment, allocation, and study completion is presented in Figure 1. Attendance and adherence in the intervention group are detailed in Supplementary Table S4.

**Table 1:** Demographic characteristics of the intervention and control groups (*N* = 88).

Variable	Category	Intervention group, *n* (%)	Control group, *n* (%)	χ^2^	df	*p*
Grade				0.144	3	0.986
	First year	18 (40.9)	18 (40.9)			
	Second year	12 (27.3)	12 (27.3)			
	Third year	7 (15.9)	8 (18.2)			
	Fourth year	7 (15.9)	6 (13.6)			
Place of origin				0.741	1	0.389
	Township	21 (47.7)	17 (38.6)			
	City	23 (52.3)	27 (61.4)			
Only-child status				0.786	1	0.375
	Yes	14 (31.8)	18 (40.9)			
	No	30 (68.2)	26 (59.1)			

**Figure 1:**
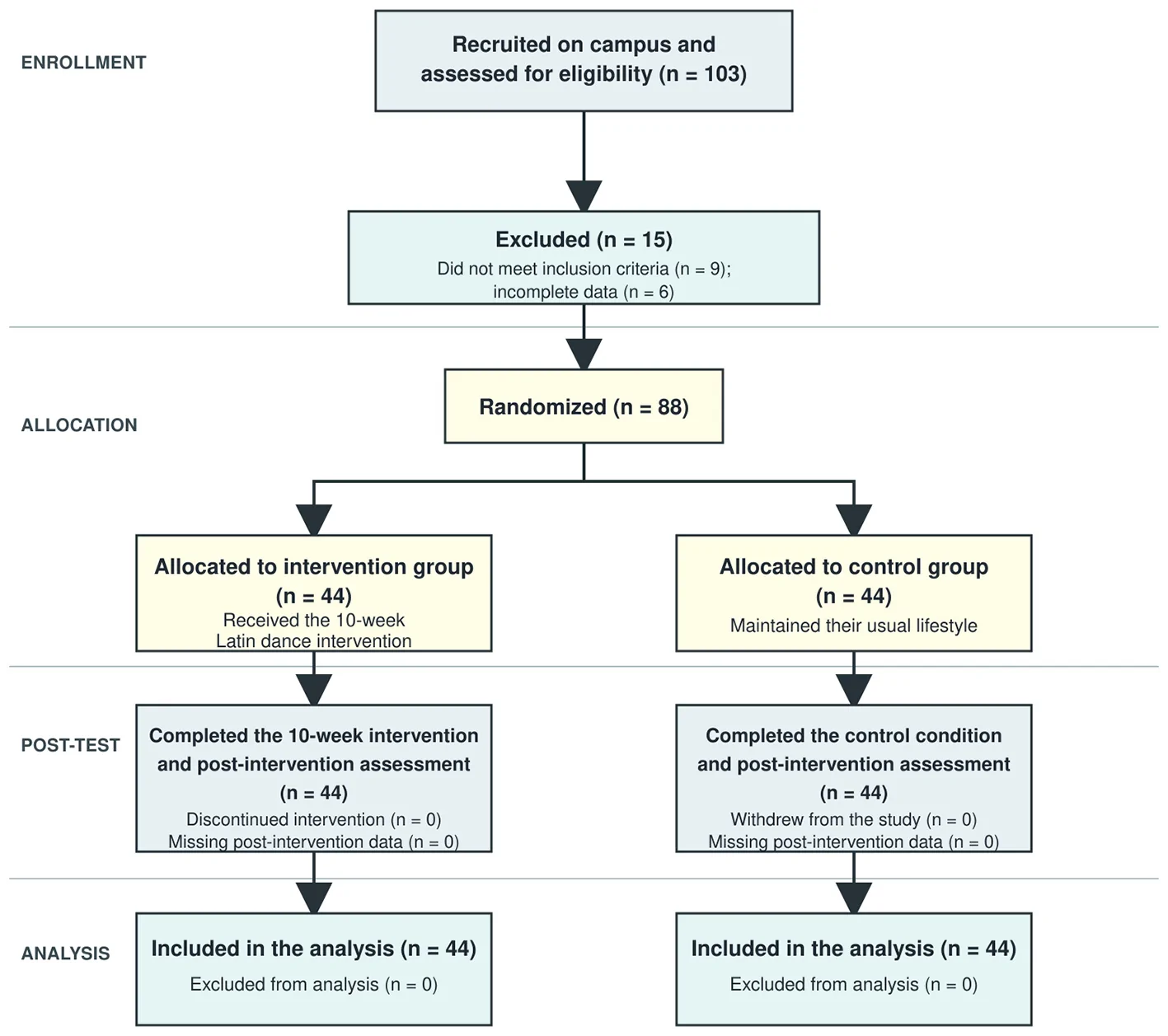
Participant flow diagram.

### 2.3 Study design and procedures

#### 2.3.1 Study design

This study employed a 2 (group: intervention vs. control) × 2 (time: baseline vs. post-intervention) mixed factorial design. Group was the between-subjects factor, and time was the within-subjects factor. The intervention group participated in a 10-week Latin dance exercise intervention. The control group received no structured exercise, psychological intervention, attention-matched sessions, or organized social contact from the study team and was asked to maintain its usual routines. Because of the behavioral nature of the intervention, participants and instructors could not be blinded to group assignment. The researcher supervising questionnaire administration was aware of group allocation. Body image and social anxiety were assessed in both groups at baseline and immediately after the intervention. To minimize temporal and environmental variation across sessions, the same trained instructor delivered all intervention sessions in the same venue, following a standardized instructional plan and schedule.

#### 2.3.2 Intervention content

The intervention consisted of Level 1 bronze-level solo routines in Cha-Cha and Rumba developed by the Chinese DanceSport Federation. Using a progressive instructional approach, the program comprised five stages: dance fundamentals, basic Cha-Cha steps, basic Rumba steps, routine consolidation, and final assessment/performance. The intervention lasted 10 weeks and included two 90-min sessions per week. Each session consisted of a warm-up, a main instructional phase, and a cool-down. The warm-up, main instructional phase, and cool-down focused on physical preparation, Latin dance technique and routine instruction, and relaxation and review of session content, respectively. A detailed overview of the intervention stages and session content is provided in Supplementary Table S3.

#### 2.3.3 Study procedures

Preparation phase: The measurement instruments were selected, and the intervention content and session schedule were developed. Participants were informed about the study purpose, intervention procedures, and relevant safety precautions before providing informed consent. A pilot study was conducted to evaluate the feasibility of the protocol and refine the study procedures.

Baseline assessment: Participants completed a demographic questionnaire and measures of body image and social anxiety to provide baseline data. They were subsequently randomly allocated in a 1:1 ratio to the intervention or control group, with random numbers generated using the RAND function in Microsoft Excel, after which baseline comparability between the groups was assessed.

Intervention phase: The intervention group participated in the 10-week Latin dance program, whereas participants in the control group maintained their usual routines. Attendance among participants in the intervention group and extracurricular physical activity in both groups were monitored throughout the intervention. An attendance rate of at least 80% was considered indicative of adequate participant adherence. Intervention fidelity, extracurricular physical activity, and adverse events were also monitored throughout the study (Supplementary Table S5).

Post-intervention assessment: After the intervention, both groups completed the same body image and social anxiety measures under conditions consistent with those used at baseline. The resulting data were then analyzed statistically.

#### 2.3.4 Measures

Body image: Body image was assessed using five appearance-related subscales of the Chinese version of the Multidimensional Body-Self Relations Questionnaire (MBSRQ). The original questionnaire was developed by Cash and colleagues (Brown et al., 1990; Cash, 2000) and adapted into Chinese by Wang and Wang (2004). Five subscales relevant to appearance-related evaluation, investment, satisfaction, and weight-related concern were selected: Overweight Preoccupation, Self-Classified Weight, Appearance Evaluation, Appearance Orientation, and Body Areas Satisfaction. Together, these subscales comprised 33 items.

The five subscales were interpreted separately. Higher Appearance Evaluation and Body Areas Satisfaction scores indicated more favorable appearance evaluation and greater body-area satisfaction, respectively. Higher Overweight Preoccupation scores indicated greater weight-related concern. Appearance Orientation reflected investment in appearance, whereas Self-Classified Weight reflected perceived weight classification. Therefore, neither direction of change in these latter two subscales was interpreted as uniformly favorable.

Items were rated on a 5-point response scale, with higher scores indicating higher levels of the construct assessed by each subscale. Relevant items in the Appearance Evaluation and Appearance Orientation subscales were reverse-scored in accordance with the instrument's scoring procedure. For the indirect-effect analysis, an aggregate body image index was calculated as the unweighted mean of the five subscale mean scores in their original scoring directions. The index was used as an aggregate analytical variable, while substantive interpretation remained based on the separate subscales. At baseline, Cronbach's α coefficients ranged from 0.857 to 0.959 across the five subscales, indicating good internal consistency (Supplementary Table S1).

Social anxiety: The Social Avoidance and Distress Scale (SAD), developed by Watson and Friend (1969), was used to assess social avoidance and distress in social interactions. The scale comprises two 14-item subscales: Social Avoidance and Social Distress.

Although the original SAD uses a dichotomous response format, the present study employed a 4-point Likert-type scale ranging from 1 = strongly disagree to 4 = strongly agree to obtain graded responses without a neutral midpoint. Following reverse scoring of the relevant items, mean scores were calculated separately for the Social Avoidance and Social Distress subscales, and an overall mean score was calculated across all 28 items. Higher scores indicated greater social avoidance and distress. Because the response format was modified, the dimensional structure of the scale was examined using principal component analysis with varimax rotation. Components with eigenvalues greater than 1 were retained, and the scree plot was examined as a complementary criterion. The Kaiser-Meyer-Olkin measure was 0.926, and Bartlett's test of sphericity was statistically significant, χ^2^(378) = 1,956.064, *p* < 0.001. Both retention criteria supported a two-component solution, which accounted for 63.33% of the total variance after rotation (Supplementary Table S2). At baseline, Cronbach's α coefficients were 0.955 for Social Avoidance, 0.953 for Social Distress, and 0.967 for the overall mean score (Supplementary Table S1), indicating good internal consistency.

### 2.4 Statistical analysis

Data were entered in Microsoft Excel and analyzed using IBM SPSS Statistics 27.0 (IBM Corp., Armonk, NY, USA). Continuous variables were presented as means and standard deviations (M ± SD), whereas categorical variables were presented as frequencies and percentages. Baseline characteristics were compared between the two groups using independent-samples *t* tests for continuous variables and χ^2^ tests for categorical variables. All tests were two-sided, with statistical significance evaluated at α = 0.05 unless otherwise specified. All 88 randomized participants were analyzed according to their originally assigned groups, with no post-randomization exclusions or missing post-intervention outcome data; thus, the analyses were consistent with the intention-to-treat principle.

The primary analyses of the body image and social anxiety outcomes were baseline-adjusted analyses of covariance (ANCOVAs). Separate ANCOVAs were conducted for the five body image subscales, the aggregate body image index, and the three social anxiety measures. In each model, the post-intervention score was specified as the dependent variable, group as the fixed factor, and the corresponding baseline score as the covariate. Homogeneity of regression slopes was evaluated using the Group × baseline-score interaction, and homogeneity of error variances was assessed using Levene's test. When the Group × baseline-score interaction was not statistically significant, the interaction term was removed before fitting the final model. Adjusted group means, adjusted mean differences with 95% confidence intervals, *F* statistics, *p* values, and partial eta squared (η_*p*_^2^) were reported. Bonferroni correction was applied separately to the six body image outcomes and the three social anxiety outcomes, resulting in adjusted significance thresholds of α = 0.0083 and α = 0.0167, respectively.

Secondary analyses examined differential change over time using a 2 (group: intervention vs. control) × 2 (time: baseline vs. post-intervention) mixed-design ANOVA for each outcome. The Group × Time interactions were interpreted as secondary evidence concerning between-group differences in change. The same Bonferroni-adjusted thresholds were applied within the body image and social anxiety outcome families. Exploratory simple-effects analyses were also reported, with unadjusted two-sided *p* values evaluated at α = 0.05.

Pearson correlations between post-intervention body image and social anxiety measures were calculated using pooled data from both groups. A statistical indirect-effect analysis was conducted using Model 4 of the PROCESS macro. Group was coded as 0 for the control group and 1 for the Latin dance group and entered as the independent variable. The post-intervention aggregate body image index was entered as the mediator, and the post-intervention Social Avoidance and Distress overall mean score was entered as the outcome. The model adjusted for the baseline aggregate body image index, baseline Social Avoidance and Distress overall mean score, grade, place of origin, and only-child status. The indirect effect and its 95% confidence interval were estimated using 5,000 bootstrap resamples. The indirect effect was considered statistically significant if the bootstrap 95% confidence interval excluded zero. Accordingly, the analysis was interpreted as an exploratory statistical indirect-effect analysis rather than a test of causal mediation.

## 3 Results

### 3.1 Assessment of common method bias

Harman's single-factor test was conducted using an unrotated principal component analysis of all self-report measurement items. The analysis yielded eight components with eigenvalues greater than 1. The first component accounted for 32.33% of the total variance, which was below the conventional 40% threshold. No single component accounted for the majority of the variance. Together, these results indicated that potential common method bias was unlikely to pose a serious threat in this study.

### 3.2 Baseline comparisons between the intervention and control groups

To assess baseline comparability, independent-samples *t* tests were used to compare baseline body image and social anxiety scores between the intervention and control groups of female college students. No statistically significant between-group differences were observed for any measure (all *p* > 0.05), and the 95% confidence intervals for all mean differences included zero (Table 2).

**Table 2:** Baseline comparisons of body image and social anxiety scores between the intervention and control groups (M ± SD).

Measure	Intervention group (*n* = 44)	Control group (*n* = 44)	*t*	*p*	95% CI for the mean difference
Overweight preoccupation	3.16 ± 0.56	2.96 ± 0.61	1.58	0.118	[−0.05, 0.45]
Self-classified weight	3.09 ± 0.60	2.95 ± 0.66	1.01	0.316	[−0.13, 0.40]
Appearance evaluation	3.09 ± 0.59	2.91 ± 0.58	1.43	0.157	[−0.07, 0.43]
Appearance orientation	3.10 ± 0.52	3.04 ± 0.55	0.53	0.598	[−0.17, 0.29]
Body areas satisfaction	3.08 ± 0.47	2.90 ± 0.61	1.54	0.128	[−0.05, 0.41]
Aggregate body image index	3.10 ± 0.48	2.95 ± 0.54	1.37	0.174	[−0.07, 0.37]
Social avoidance	2.86 ± 0.44	3.03 ± 0.49	−1.76	0.082	[−0.37, 0.02]
Social distress	2.95 ± 0.35	3.09 ± 0.53	−1.46	0.149	[−0.33, 0.05]
Social avoidance and distress overall mean score	2.91 ± 0.36	3.05 ± 0.47	−1.66	0.101	[−0.32, 0.03]

### 3.3 Effects of the Latin dance exercise intervention on body image in female college students

The assumptions of homogeneity of regression slopes and error variances were satisfied for all ANCOVA models (all *p* > 0.05). Primary baseline-adjusted ANCOVAs showed that, compared with the control group, the Latin dance group had lower adjusted post-intervention scores for Overweight Preoccupation and Self-Classified Weight and higher adjusted scores for Appearance Evaluation, Body Areas Satisfaction, and the aggregate body image index. These group differences were statistically significant, *F*(1, 85) = 54.17–130.68, all *p* < 0.001, η_*p*_^2^ = 0.389–0.606. No adjusted group difference was observed for Appearance Orientation, *F*(1, 85) = 0.15, *p* = 0.700, η_*p*_^2^ = 0.002. All five statistically significant results met the Bonferroni-adjusted threshold of α = 0.0083 (Table 3).

**Table 3:** Baseline-adjusted ANCOVA results for the body image measures.

Measure	Latin dance group adjusted *M* (*SE*)	Control group adjusted *M (SE*)	Adjusted mean difference	95% CI	*F*(1, 85)	*p*	η_*p*_^2^
Overweight preoccupation	2.323 (0.052)	2.889 (0.052)	−0.566	[−0.713, −0.419]	58.53	< 0.001	0.408
Self-classified weight	2.380 (0.061)	3.018 (0.061)	−0.638	[−0.810, −0.465]	54.17	< 0.001	0.389
Appearance evaluation	4.495 (0.091)	3.164 (0.091)	1.331	[1.075, 1.588]	106.43	< 0.001	0.556
Appearance orientation	2.987 (0.031)	3.004 (0.031)	−0.017	[−0.104, 0.070]	0.15	0.700	0.002
Body areas satisfaction	4.489 (0.084)	3.120 (0.084)	1.370	[1.131, 1.608]	130.68	< 0.001	0.606
Aggregate body image index	3.334 (0.024)	3.039 (0.024)	0.295	[0.228, 0.362]	76.82	< 0.001	0.475

Values are baseline-adjusted estimated marginal means (*SE*). Adjusted mean differences were calculated as the Latin dance group minus the control group. The Bonferroni-adjusted significance threshold was α = 0.0083. CI, confidence interval; SE, standard error; η_*p*_^2^, partial eta squared.

Secondary mixed-design ANOVAs showed statistically significant Group × Time interactions for Overweight Preoccupation, Self-Classified Weight, Appearance Evaluation, and Body Areas Satisfaction, *F*(1, 86) = 25.95–48.55, all *p* < 0.001, η_*p*_^2^ = 0.23–0.36. The interactions were not statistically significant for Appearance Orientation, *F*(1, 86) = 0.41, *p* = 0.526, η_*p*_^2^ = 0.01, or the aggregate body image index, *F*(1, 86) = 1.68, *p* = 0.198, η_*p*_^2^ = 0.02. Complete mixed-design ANOVA and exploratory simple-effects results are provided in Supplementary Table S6.

Exploratory simple-effects analyses indicated that Overweight Preoccupation and Self-Classified Weight decreased from baseline to post-intervention in the Latin dance group, whereas Appearance Evaluation and Body Areas Satisfaction increased, all *p* < 0.001. At post-intervention, the Latin dance group also showed lower scores on Overweight Preoccupation and Self-Classified Weight and higher scores on Appearance Evaluation and Body Areas Satisfaction than the control group, all *p* < 0.001.

### 3.4 Effects of the Latin dance intervention on social anxiety in female college students

Primary baseline-adjusted ANCOVAs showed that the Latin dance group had lower adjusted post-intervention scores than the control group for Social Avoidance, Social Distress, and the Social Avoidance and Distress overall mean score, *F*(1, 85) = 23.72–32.85, all *p* < 0.001, η_*p*_^2^ = 0.218–0.279. The adjusted mean differences ranged from −0.515 to −0.482, and all 95% confidence intervals excluded zero. All three results met the Bonferroni-adjusted significance threshold of α = 0.0167 (Table 4).

**Table 4:** Baseline-adjusted ANCOVA results for the social anxiety measures.

Measure	Latin dance group adjusted *M (SE)*	Control group adjusted *M (SE)*	Adjusted mean difference	95% CI	*F*(1, 85)	*p*	$\eta_{p}^{2}$
Social avoidance	2.705 (0.069)	3.186 (0.069)	−0.482	[−0.678, −0.285]	23.72	< 0.001	0.218
Social distress	2.747 (0.063)	3.261 (0.063)	−0.515	[−0.693, −0.336]	32.85	< 0.001	0.279
Social avoidance and distress overall mean score	2.723 (0.062)	3.227 (0.062)	−0.504	[−0.681, −0.327]	32.11	< 0.001	0.274

Values are baseline-adjusted estimated marginal means (*SE*). Adjusted mean differences were calculated as the Latin dance group minus the control group. The Bonferroni-adjusted significance threshold was α = 0.0167. CI, confidence interval; SE, standard error; η_*p*_^2^, partial eta squared.

Secondary mixed-design ANOVAs showed statistically significant Group × Time interactions for Social Avoidance, Social Distress, and the Social Avoidance and Distress overall mean score, *F*(1, 86) = 5.98–7.64, *p* = 0.007–0.017, η_*p*_^2^ = 0.07–0.08. Based on the exact unrounded *p* value of 0.016534, the Social Avoidance interaction also met the Bonferroni-adjusted threshold of α = 0.0167. Complete mixed-design ANOVA and exploratory simple-effects results are provided in Supplementary Table S7.

Exploratory between-group simple-effects analyses at each time point showed no statistically significant baseline differences for any social anxiety measure (*F* = 2.13–3.09, *p* = 0.082–0.148, η_*p*_^2^ = 0.02–0.04). At post-intervention, all three scores were significantly lower in the Latin dance group than in the control group (*F* = 26.37–33.43, all *p* < 0.001, η_*p*_^2^ = 0.24–0.28).

Exploratory within-group simple-effects analyses showed that Social Distress (*F* = 4.35, *p* = 0.040, η_*p*_^2^ = 0.05) and the Social Avoidance and Distress overall mean score (*F* = 4.04, *p* = 0.048, η_*p*_^2^ = 0.05) decreased from baseline to post-intervention in the Latin dance group. The change in Social Avoidance was not statistically significant (*F* = 2.92, *p* = 0.092, η_*p*_^2^ = 0.03). None of the baseline-to-post-intervention changes in the control group were statistically significant (*F* = 3.08–3.61, *p* = 0.061–0.083, η_*p*_^2^ = 0.04).

No apparent adverse events were observed or recorded during the intervention (Supplementary Table S5).

### 3.5 Correlations between body image and social anxiety

Table 5 presents Pearson correlations based on the pooled post-intervention data (*N* = 88). Overweight Preoccupation, Self-Classified Weight, and Appearance Orientation were positively correlated with Social Avoidance, Social Distress, and the Social Avoidance and Distress overall mean score (*r* = 0.25–0.65, all *p* < 0.05). In contrast, Appearance Evaluation, Body Areas Satisfaction, and the aggregate body image index were negatively correlated with all three social anxiety measures (*r* = −0.81 to −0.61, all *p* < 0.01). Because the data from both groups were pooled, these correlations were interpreted descriptively.

**Table 5:** Correlation analysis between body image and social anxiety measures (*N* = 88).

Variable	1	2	3	4	5	6	7	8	9
1. Overweight preoccupation	1								
2. Self-classified weight	0.549^**^	1							
3. Appearance evaluation	−0.719^**^	−0.752^**^	1						
4. Appearance orientation	0.127	0.205	−0.212^*^	1					
5. Body areas satisfaction	−0.732^**^	−0.756^**^	0.924^**^	−0.242^*^	1				
6. Aggregate body image index	−0.496^**^	−0.499^**^	0.888^**^	−0.031	0.874^**^	1			
7. Social avoidance	0.622^**^	0.632^**^	−0.724^**^	0.334^**^	−0.770^**^	−0.608^**^	1		
8. Social distress	0.626^**^	0.601^**^	−0.775^**^	0.249^*^	−0.776^**^	−0.683^**^	0.823^**^	1	
9. Social avoidance and distress overall mean score	0.654^**^	0.647^**^	−0.785^**^	0.307^**^	−0.810^**^	−0.675^**^	0.958^**^	0.952^**^	1

^*^p < 0.05, ^**^ *p* < 0.01.

### 3.6 Indirect effect of the Latin dance intervention on social anxiety through body image in female college students

Given the non-significant secondary Group × Time interaction for the aggregate body image index, the indirect-effect analysis was considered exploratory. A baseline-adjusted regression model was estimated using Model 4 of the PROCESS macro. The analysis adjusted for the baseline aggregate body image index, baseline Social Avoidance and Distress overall mean score, grade, place of origin, and only-child status. Group was coded as 0 for the control group and 1 for the Latin dance group. The post-intervention aggregate body image index served as the mediator, and the post-intervention Social Avoidance and Distress overall mean score served as the outcome.

The model predicting the post-intervention aggregate body image index was statistically significant (*R* = 0.70, *R*^2^ = 0.50, *F*(6, 81) = 13.64, *p* < 0.001). After adjustment for the covariates, group significantly predicted the post-intervention aggregate body image index (*B* = 0.30, *t*(81) = 8.74, *p* < 0.001, 95% CI [0.24, 0.37]). The primary baseline-adjusted ANCOVA showed that the Latin dance group had a higher adjusted post-intervention aggregate body image index than the control group, *F*(1, 85) = 76.82, *p* < 0.001, η_*p*_^2^ = 0.475, with an adjusted mean difference of 0.295, 95% CI [0.228, 0.362] (Table 3). By contrast, the secondary Group × Time interaction for this index was not statistically significant, *F*(1, 86) = 1.68, *p* = 0.198, η_*p*_^2^ = 0.02 (Supplementary Table S6). These analyses addressed related but distinct contrasts: the ANCOVA evaluated the baseline-adjusted post-intervention group difference, whereas the Group × Time interaction evaluated the between-group difference in change over time. Accordingly, the significant ANCOVA result did not establish a differential change in the aggregate body image index, and the indirect-effect findings remained exploratory.

The model predicting the post-intervention Social Avoidance and Distress overall mean score was also statistically significant (*R* = 0.71, *R*^2^ = 0.50, *F*(7, 80) = 11.64, *p* < 0.001). The post-intervention aggregate body image index was negatively associated with the post-intervention Social Avoidance and Distress overall mean score (*B* = −1.40, *t*(80) = −5.63, *p* < 0.001, 95% CI [−1.89, −0.90]). With the mediator and covariates included in the model, the direct effect of group was not statistically significant (*B* = −0.08, *t*(80) = −0.76, *p* = 0.449, 95% CI [−0.30, 0.13]; Table 6 and Figure 2).

**Table 6:** Regression models linking the Latin dance intervention, body image, and social anxiety (*N* = 88).

Outcome/predictor	*R*	*R* ^2^	*F*	*B*	*t*	LLCI	ULCI
**Aggregate body image index**	**0.70**	**0.50**	**13.64** ^***^				
Group				0.30	8.74^***^	0.24	0.37
Grade				0.01	0.34	−0.03	0.04
Place of origin				0.05	1.37	−0.02	0.12
Only-child status				0.02	0.59	−0.05	0.09
Baseline aggregate body image index				0.01	0.25	−0.06	0.08
Baseline social avoidance and distress overall mean score				0.04	1.05	−0.04	0.12
**Social avoidance and distress overall mean score**	**0.71**	**0.50**	**11.64** ^***^				
Group				−0.08	−0.76	−0.30	0.13
Grade				0.08	2.33^*^	0.01	0.15
Place of origin				0.09	1.21	−0.06	0.25
Only-child status				0.03	0.43	−0.12	0.19
Post-intervention aggregate body image index				−1.40	−5.63^***^	−1.89	−0.90
Baseline aggregate body image index				0.05	0.61	−0.10	0.20
Baseline social avoidance and distress overall mean score				0.05	0.59	−0.13	0.24

B denotes the unstandardized regression coefficient; LLCI and ULCI denote the lower and upper limits of the 95% confidence interval, respectively. Bold rows indicate the outcome variable of each regression model, and the *R, R*^2^, and *F* values in these rows refer to the overall model. ^*^ *p* < 0.05, ^***^ *p* < 0.001.

**Figure 2:**
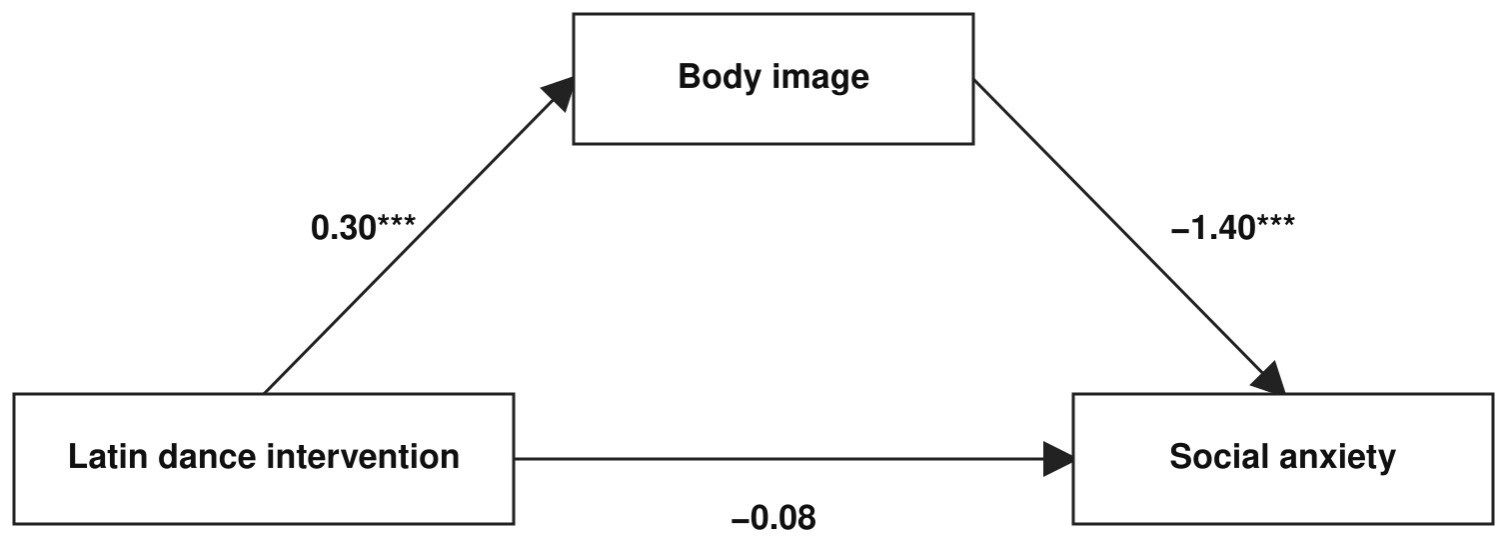
Statistical indirect-effect model of body image in the relationship between the Latin dance intervention and social anxiety. ^***^ *p* < 0.001.

As shown in Table 7, the indirect effect of group on the Social Avoidance and Distress overall mean score through the aggregate body image index was −0.43. The bootstrap 95% CI [−0.61, −0.26] did not include zero, indicating a statistically significant indirect effect. The direct effect was −0.08, with a 95% CI [−0.30, 0.13] that included zero and was therefore not statistically significant. The total effect was −0.51, with a 95% CI [−0.73, −0.29] that excluded zero. Taken together, these results were consistent with an exploratory statistical indirect effect involving the aggregate body image index.

**Table 7:** Indirect-effect analysis (*N* = 88).

Effect	Estimate	*SE*	LLCI	ULCI	Proportion (%)
Total effect	−0.51	0.11	−0.73	−0.29	100
Direct effect	−0.08	0.11	−0.30	0.13	16
Indirect effect through body image	−0.43	0.09	−0.61	−0.26	84

*SE* denotes standard error; LLCI and ULCI denote the lower and upper limits of the 95% confidence interval, respectively. Bootstrap estimates based on 5,000 resamples are reported for the indirect effect.

## 4 Discussion

Primary baseline-adjusted ANCOVAs showed that, compared with the usual-activity control group, the Latin dance group had lower adjusted post-intervention Overweight Preoccupation scores and lower scores on all three social anxiety measures. The group also had higher adjusted Appearance Evaluation and Body Areas Satisfaction scores. Self-Classified Weight was lower in the Latin dance group, but this difference was interpreted descriptively because the subscale does not have a uniformly favorable direction. The adjusted aggregate body image index was also higher, whereas no adjusted group difference was observed for Appearance Orientation. Taken together, these results partially supported H1 and supported H2. Secondary Group × Time analyses were generally consistent with the primary findings for the separate body image and social anxiety measures. However, the interaction for the aggregate body image index was not statistically significant.

Further analyses using a baseline-adjusted model identified a statistically significant indirect effect involving the aggregate body image index. With the aggregate index and covariates included in the model, the direct effect of group assignment was not statistically significant. This pattern was consistent with H3 but provided only preliminary evidence of a statistical indirect association.

Regarding body image, the baseline-adjusted ANCOVAs showed that the Latin dance group had lower adjusted post-intervention Overweight Preoccupation and Self-Classified Weight scores and higher adjusted Appearance Evaluation, Body Areas Satisfaction, and aggregate body image index scores than the control group. No adjusted group difference was observed for Appearance Orientation. The differences in Self-Classified Weight and the aggregate body image index were interpreted descriptively rather than as uniformly favorable. The Group × Time analyses identified differential changes in Overweight Preoccupation, Self-Classified Weight, Appearance Evaluation, and Body Areas Satisfaction, but not in Appearance Orientation or the aggregate body image index. Overall, the findings across multiple body image dimensions are broadly consistent with Hu et al. (2025), who reported beneficial effects of Latin dance on body esteem.

From the perspective of social comparison theory, women exposed to thin-ideal beauty standards are more likely to engage in upward appearance comparisons, which may increase weight-related concerns (Festinger, 1954; Heinberg and Thompson, 1992). Repeated Latin dance practice emphasizes bodily control and self-expression, thereby foregrounding body functionality and movement competence rather than external scrutiny. This emphasis may offer an alternative to upward appearance-comparison processes. Targeted exercises involving the waist and abdominal regions may also provide opportunities for readily perceived positive bodily feedback. Together with the social interaction embedded in group practice, these experiences may have contributed to reduced Overweight Preoccupation and improved Appearance Evaluation and Body Areas Satisfaction. This interpretation is broadly consistent with conceptualizations of positive body image that emphasize body acceptance, appreciation, and respect (Tylka and Wood-Barcalow, 2015). It also aligns with grounded cognition theory, which holds that bodily experiences and situated actions influence self-evaluation and emotional states (Barsalou, 2008).

By contrast, Appearance Orientation showed no statistically significant Group × Time interaction. Because the study did not assess the attentional or cognitive processes potentially related to this dimension, the available data do not support a specific explanation for this finding. Further research is needed to clarify the conditions under which Appearance Orientation may change.

Regarding social anxiety, the baseline-adjusted ANCOVAs showed that the Latin dance group had lower adjusted post-intervention Social Avoidance, Social Distress, and Social Avoidance and Distress overall mean scores than the control group. The Group × Time analyses provided generally consistent evidence of between-group differences in change over time. These findings are broadly consistent with Li et al. (2025), who reported that a dance intervention reduced social anxiety. The distinctive characteristics of Latin dance may offer one possible interpretation of this pattern. Compared with exercise forms that place greater emphasis on physical exertion, such as running and aerobics, Latin dance emphasizes bodily expressiveness and emotional communication. Latin dance practice requires participants to perform expressive bodily movements in the presence of others. This setting resembles the social-evaluative situations that individuals with social anxiety often fear, particularly situations involving the possibility that their performance will be negatively judged. Repeated practice gives participants opportunities to become more comfortable with expressive performance and to encounter positive experiences that challenge anticipated negative evaluation. Emotional arousal elicited by musical rhythm and the sense of achievement associated with skill development may provide additional sources of positive reinforcement.

This interpretation is compatible with the cognitive-behavioral model of anxiety in social phobia proposed by Rapee and Heimberg (1997). The model emphasizes biased processing of information concerning potential social evaluation. Within this framework, repeated positive social experiences may help revise negative expectations by disconfirming anticipated evaluations, thereby reducing avoidance and emotional distress. This process also offers a route to broader social adjustment. The present findings are also consistent with those of Wang et al. (2009) regarding group exercise interventions for social anxiety.

These explanations remain tentative because the study assessed body image and social anxiety outcomes but did not directly measure self-objectification, body surveillance, attentional bias, body functionality, or social-evaluative expectations. The theories discussed above therefore provide context for interpreting the findings rather than mechanisms directly tested in this study.

Descriptive analyses of the pooled post-intervention data showed associations between the body image and social anxiety measures, consistent with previous evidence linking body image to social anxiety (Gan and Jiang, 2024). Within the baseline-adjusted model, the statistical indirect effect of the Latin dance intervention on the Social Avoidance and Distress overall mean score via the aggregate body image index was statistically significant. The corresponding direct effect was not statistically significant. This pattern is consistent with the proposed statistical indirect pathway.

Two interrelated psychological functions of body image provide a framework for interpreting this pattern. Body image not only provides a cognitive basis for evaluating one's appearance but also serves as a reference point for anticipating others' evaluations and assessing whether social situations are safe. For female college students, the body is not merely an object of appearance evaluation; it is also closely tied to confident self-presentation in interpersonal interactions. Individuals who evaluate their bodies negatively are more likely to interpret others' attention as scrutiny or criticism and consequently experience heightened self-focused attention and rumination (Bu and Ma, 2024). They may then use avoidance or concealment to reduce the resulting discomfort. Although such strategies provide temporary relief, they reinforce negative beliefs and thereby maintain social anxiety over time (Wells et al., 1995). This interpretation is consistent with the findings of Jiang and Zhang (2024) and Zhao et al. (2024). Conversely, a more positive body image has been associated with lower levels of social anxiety (Singh and Tripathi, 2025). Individuals with a more positive body image tend to experience fewer negative appearance-related cognitions and show better social adjustment.

Objectification theory may also help interpret the findings (Zheng et al., 2015). Nevertheless, whether self-objectification or body surveillance changed during the intervention remains unclear because neither process was assessed.

Taken together, the pattern of findings is consistent with a possible pathway linking Latin dance exercise to lower social anxiety among female college students. The observed statistical indirect effect may partly involve changes in participants' relationships with their bodies. This interpretation may inform the design of university mental health programs and physical education curricula. Such initiatives may benefit from considering not only the physiological benefits of exercise but also the bodily experiences it creates. Strategies that promote positive bodily experiences and reframe body-related cognitions may strengthen appreciation of body functionality and, in turn, support better social adjustment.

Several limitations should be considered when interpreting these findings. The study used a convenience sample from a single university and did not employ formal allocation concealment, which may have introduced selection bias. The absence of prospective trial registration limits independent verification of whether the reported outcomes and analyses were prespecified and should therefore be considered when interpreting the findings. Participants, instructors, and the researcher supervising questionnaire administration were not blinded to group assignment. The passive usual-activity control group did not receive study-provided attention, organized social contact, or structured physical activity comparable to those received by the intervention group, and expectancy effects were not controlled. These design features may have introduced expectancy or measurement bias and may have affected the magnitude of the observed between-group differences, thereby limiting conclusions about effects specific to Latin dance. The absence of follow-up prevented evaluation of whether the observed changes were maintained over time; accordingly, their durability remains uncertain. The statistical indirect-effect finding should be considered preliminary because of the modest sample size and the design limitations of the statistical indirect-effect analysis. Although the primary ANCOVA identified an adjusted post-intervention group difference in the aggregate body image index, the corresponding secondary Group × Time interaction was not statistically significant. Because this index also combined subscales with different substantive meanings, findings based on the aggregate index should be interpreted cautiously. Finally, the SAD response format was changed from dichotomous to four-point Likert responses. Although the modified format showed high internal consistency and an exploratory two-factor solution, its factor structure and construct validity require confirmation in larger and more diverse samples.

Future randomized intervention studies should be prospectively registered in a publicly accessible trial registry before participant enrollment, with outcomes and analysis plans clearly prespecified. They should also use formal allocation concealment, include at least three assessment waves, and employ attention-matched active control conditions, such as aerobic exercise or non-expressive group exercise.

## 5 Conclusion

Baseline-adjusted analyses showed that, compared with usual activity, assignment to the Latin dance intervention was associated with lower adjusted post-intervention Overweight Preoccupation and social anxiety scores and higher adjusted post-intervention Appearance Evaluation and Body Areas Satisfaction scores. Group × Time analyses provided generally consistent evidence of between-group differences in change over time. Within the baseline-adjusted model, the statistically significant indirect effect provided preliminary evidence of an indirect association involving the aggregate body image index and lower social anxiety. These findings support further evaluation of body image as a potential intervention target in university-based group exercise programs, including Latin dance. Given the lack of allocation concealment and assessor blinding and the use of a passive control group, causal interpretation of these findings should remain tentative.

## Data Availability

The raw data supporting the conclusions of this article will be made available by the authors, without undue reservation.
