# Supplementary material for: Effects of Latin dance exercise on body image and social anxiety in female college students
Source: Front Psychol. 2026 Oct 9;17:1958896. doi: 10.3389/fpsyg.2026.1958896 (PMC13690543; doi:10.3389/fpsyg.2026.1958896)
Supplement: Supplementary file 1 [file Table_1.docx]

**Supplementary Material for: Effects of Latin Dance Exercise on Body Image and Social Anxiety in Female College Students**

**Supplementary Table S1.** Internal consistency of the body image and social anxiety measures at baseline (N = 88)

| **Measure** | **Subscale or score** | **No. of items** | **Cronbach's α** |
| --- | --- | --- | --- |
| Chinese Multidimensional Body-Self Relations Questionnaire | Overweight Preoccupation | 3 | 0.857 |
|  | Self-Classified Weight | 2 | 0.858 |
|  | Appearance Evaluation | 7 | 0.936 |
|  | Appearance Orientation | 12 | 0.959 |
|  | Body Areas Satisfaction | 9 | 0.942 |
|  | Aggregate body image index | 33 | 0.978 |
| Social Avoidance and Distress Scale | Social Avoidance | 14 | 0.955 |
|  | Social Distress | 14 | 0.953 |
|  | Social Avoidance and Distress overall mean score | 28 | 0.967 |

**Supplementary Table S2.** Exploratory analysis of the factor structure of the modified Social Avoidance and Distress Scale (N = 88)

| **Item** | **Factor 1** | **Factor 2** | **Communality** |
| --- | --- | --- | --- |
| t2 | 0.692 |  | 0.63 |
| t4 | 0.733 |  | 0.613 |
| t8 | 0.761 |  | 0.651 |
| t9 | 0.715 |  | 0.592 |
| t13 | 0.691 |  | 0.585 |
| t17 | 0.772 |  | 0.696 |
| t18 | 0.687 |  | 0.567 |
| t19 | 0.74 |  | 0.631 |
| t21 | 0.803 |  | 0.671 |
| t22 | 0.711 |  | 0.605 |
| t24 | 0.803 |  | 0.709 |
| t25 | 0.725 |  | 0.633 |
| t26 | 0.764 |  | 0.649 |
| t27 | 0.783 |  | 0.685 |
| t1 |  | 0.791 | 0.711 |
| t3 |  | 0.715 | 0.597 |
| t5 |  | 0.703 | 0.624 |
| t6 |  | 0.819 | 0.752 |
| t7 |  | 0.694 | 0.628 |
| t10 |  | 0.746 | 0.589 |
| t11 |  | 0.787 | 0.725 |
| t12 |  | 0.581 | 0.516 |
| t14 |  | 0.701 | 0.551 |
| t15 |  | 0.749 | 0.628 |
| t16 |  | 0.809 | 0.721 |
| t20 |  | 0.773 | 0.674 |
| t23 |  | 0.528 | 0.461 |
| t28 |  | 0.764 | 0.636 |
| Eigenvalue | 9.071 | 8.661 |  |
| Variance explained (%) | 32.397 | 30.931 |  |
| Cumulative variance explained (%) | 32.397 | 63.329 |  |

*Note*. Principal component analysis with varimax rotation was used. The Kaiser–Meyer–Olkin measure was 0.926, and Bartlett's test of sphericity was statistically significant, χ²(378) = 1,956.064, *p* < 0.001. Factors with eigenvalues greater than 1 were retained, and the scree plot was examined as a complementary criterion. Before rotation, the first and second components had eigenvalues of 14.862 and 2.870 and explained 53.08% and 10.25% of the variance, respectively. After rotation, they explained 32.397% and 30.931% of the variance, respectively, for a cumulative explained variance of 63.329%. Factor 1 corresponded to Social Avoidance, and Factor 2 corresponded to Social Distress. Factor loadings below 0.40 are not displayed.

**Supplementary Table S3.** Ten-week Latin dance exercise intervention program

| **Training stage** | **Sessions** | **Instructional objective** | **Session structure and principal content** |
| --- | --- | --- | --- |
| 1. Latin dance fundamentals | 1 | Understand the nature and basic principles of Latin dance and establish an initial learning framework. | 1. Warm-up; 2. introduction to Latin dance categories and styles; 3. musical-rhythm perception exercises; 4. instruction in basic posture, arm positions, and foot positions; 5. cool-down and session review. |
| 2. Basic Cha-Cha steps | 6 | Become familiar with basic Cha-Cha steps and perform them to music. | 1. Warm-up; 2. in-place weight-transfer and time-step exercises; 3. forward and backward lock steps and square steps; 4. quarter-turn and hand-to-hand figures; 5. cool-down and session review. |
| 3. Basic Rumba steps | 6 | Become familiar with basic Rumba steps and perform them to music. | 1. Warm-up; 2. in-place weight-transfer and time-step exercises; 3. forward and backward steps and square steps; 4. quarter-turn and Cucaracha figures; 5. cool-down and session review. |
| 4. Step consolidation and routines | 6 | Consolidate the basic steps and learn solo Cha-Cha and Rumba routines. | 1. Warm-up; 2. review and consolidation of basic Cha-Cha and Rumba steps; 3. linked step sequences; 4. instruction and practice of solo Cha-Cha and Rumba routines; 5. cool-down and session review. |
| 5. Assessment and performance | 1 | Evaluate learning outcomes and strengthen participants' active engagement and confidence. | 1. Warm-up; 2. group-based assessment using multiple scoring approaches; 3. selected performances; 4. feedback, summary, and cool-down. |

*Note.* The program comprised 20 sessions delivered over 10 weeks, with two 90-minute sessions per week.

**Supplementary Table S4.** Intervention dose, adherence, and participant retention in the Latin dance group (N = 44)

| **Indicator** | **Value** |
| --- | --- |
| Participants in the Latin dance group | 44 |
| Intervention duration (weeks) | 10 |
| Sessions per week | 2 |
| Duration of each session (min) | 90 |
| Scheduled sessions per participant | 20 |
| Total scheduled attendances across participants | 880 |
| Adherence criterion | Attendance ≥80% (at least 16 sessions) |
| Total recorded attendances | 847 |
| Mean sessions attended per participant | 19.25 |
| Mean attendance rate (%) | 96.25 |
| Participants meeting the adherence criterion | 44 |
| Participants with at least one absence | 15 |
| Total absences | 33 |
| Participants excluded for inadequate adherence | 0 |
| Participants who withdrew during the intervention | 0 |
| Participants who completed the post-intervention assessment | 44 |
| Participants included in the final analysis | 44 |

**Supplementary Table S5.** Intervention fidelity and process monitoring

| **Component** | **Prespecified requirement** | **Monitoring method** | **Implementation** |
| --- | --- | --- | --- |
| Intervention delivery | All sessions delivered by the same instructor | Standardized scheduling and delivery throughout the intervention | All sessions were delivered by the researcher |
| Completion of instructional content | Prespecified content completed according to the planned stages | Instructional progress records | Completed as planned |
| Attendance monitoring | Attendance recorded at every session | On-site sign-in and attendance records | Completed for all sessions |
| Extracurricular activity in the Latin dance group | No additional participation in similar specialized training | Periodic inquiries and exercise logs or check-ins | No apparent deviation was identified |
| Lifestyle monitoring in the control group | Usual lifestyle maintained without newly initiated systematic exercise | Periodic inquiries and exercise logs or check-ins | No newly initiated systematic exercise was identified |
| Adverse-event monitoring | Discomfort, injury, or other unexpected events recorded | On-site observation and process records | No apparent adverse events were observed or recorded |
| Review of protocol adherence and data completeness | Assessment of inadequate adherence, key missing data, or major protocol deviations | End-of-study summary review | Both groups completed the post-intervention assessment and were included in the final analysis |

**Supplementary Table S6.** Complete mixed-design ANOVA and exploratory simple-effects results for the body image measures

| **Measure** | **Effect** | **Baseline** | **Post-intervention** | **F(1, 86)** | **p** | **ηₚ²** |
| --- | --- | --- | --- | --- | --- | --- |
| Overweight Preoccupation | Latin dance group (n = 44) | 3.16 ± 0.56 | 2.33 ± 0.36 | 76.58 | < 0.001 | 0.47 |
|  | Control group (n = 44) | 2.96 ± 0.61 | 2.88 ± 0.33 | 0.78 | 0.380 | 0.01 |
|  | Time main effect |  |  | 46.40 | < 0.001 | 0.35 |
|  | Group main effect |  |  | 5.02 | 0.028 | 0.06 |
|  | Group simple effect: baseline |  |  | 2.48 | 0.118 | 0.03 |
|  | Group simple effect: post-intervention |  |  | 54.24 | < 0.001 | 0.39 |
|  | Group × Time |  |  | 30.95 | < 0.001 | 0.27 |
| Self-Classified Weight | Latin dance group (n = 44) | 3.09 ± 0.60 | 2.39 ± 0.39 | 44.44 | < 0.001 | 0.34 |
|  | Control group (n = 44) | 2.95 ± 0.66 | 3.01 ± 0.42 | 0.29 | 0.592 | 0.00 |
|  | Time main effect |  |  | 18.78 | < 0.001 | 0.18 |
|  | Group main effect |  |  | 8.19 | 0.005 | 0.09 |
|  | Group simple effect: baseline |  |  | 1.02 | 0.316 | 0.01 |
|  | Group simple effect: post-intervention |  |  | 52.13 | < 0.001 | 0.38 |
|  | Group × Time |  |  | 25.95 | < 0.001 | 0.23 |
| Appearance Evaluation | Latin dance group (n = 44) | 3.09 ± 0.59 | 4.48 ± 0.57 | 107.74 | < 0.001 | 0.56 |
|  | Control group (n = 44) | 2.91 ± 0.58 | 3.18 ± 0.63 | 3.84 | 0.053 | 0.04 |
|  | Time main effect |  |  | 76.13 | < 0.001 | 0.47 |
|  | Group main effect |  |  | 78.98 | < 0.001 | 0.48 |
|  | Group simple effect: baseline |  |  | 2.05 | 0.157 | 0.02 |
|  | Group simple effect: post-intervention |  |  | 104.42 | < 0.001 | 0.55 |
|  | Group × Time |  |  | 35.45 | < 0.001 | 0.29 |
| Appearance Orientation | Latin dance group (n = 44) | 3.10 ± 0.52 | 2.99 ± 0.18 | 1.99 | 0.162 | 0.02 |
|  | Control group (n = 44) | 3.04 ± 0.55 | 3.00 ± 0.23 | 0.26 | 0.612 | 0.00 |
|  | Time main effect |  |  | 1.84 | 0.179 | 0.02 |
|  | Group main effect |  |  | 0.14 | 0.714 | 0.00 |
|  | Group simple effect: baseline |  |  | 0.28 | 0.598 | 0.00 |
|  | Group simple effect: post-intervention |  |  | 0.09 | 0.765 | 0.00 |
|  | Group × Time |  |  | 0.41 | 0.526 | 0.01 |
| Body Areas Satisfaction | Latin dance group (n = 44) | 3.08 ± 0.47 | 4.48 ± 0.52 | 137.53 | < 0.001 | 0.62 |
|  | Control group (n = 44) | 2.90 ± 0.61 | 3.12 ± 0.58 | 3.51 | 0.064 | 0.04 |
|  | Time main effect |  |  | 92.50 | < 0.001 | 0.52 |
|  | Group main effect |  |  | 90.59 | < 0.001 | 0.51 |
|  | Group simple effect: baseline |  |  | 2.36 | 0.128 | 0.03 |
|  | Group simple effect: post-intervention |  |  | 133.87 | < 0.001 | 0.61 |
|  | Group × Time |  |  | 48.55 | < 0.001 | 0.36 |
| Aggregate body image index | Latin dance group (n = 44) | 3.10 ± 0.48 | 3.34 ± 0.15 | 8.31 | 0.005 | 0.09 |
|  | Control group (n = 44) | 2.95 ± 0.54 | 3.04 ± 0.16 | 1.10 | 0.297 | 0.01 |
|  | Time main effect |  |  | 7.73 | 0.007 | 0.08 |
|  | Group main effect |  |  | 14.86 | < 0.001 | 0.15 |
|  | Group simple effect: baseline |  |  | 1.88 | 0.174 | 0.02 |
|  | Group simple effect: post-intervention |  |  | 80.35 | < 0.001 | 0.48 |
|  | Group × Time |  |  | 1.68 | 0.198 | 0.02 |

*Note.* Values are presented as M ± SD. The Bonferroni-adjusted α = 0.0083 applies only to the six body image Group × Time interaction tests. The simple-effects analyses were exploratory; their two-sided p values were not adjusted for multiple comparisons. ηₚ², partial eta squared.

**Supplementary Table S7.** Complete mixed-design ANOVA and exploratory simple-effects results for the social anxiety measures

| **Measure** | **Effect** | **Baseline** | **Post-intervention** | **F(1, 86)** | **p** | **ηₚ²** |
| --- | --- | --- | --- | --- | --- | --- |
| Social Avoidance | Latin dance group (n = 44) | 2.86 ± 0.44 | 2.70 ± 0.45 | 2.92 | 0.092 | 0.03 |
|  | Control group (n = 44) | 3.03 ± 0.49 | 3.19 ± 0.46 | 3.08 | 0.083 | 0.04 |
|  | Time main effect |  |  | 0.001 | 0.971 | 0.00 |
|  | Group main effect |  |  | 21.34 | < 0.001 | 0.20 |
|  | Group simple effect: baseline |  |  | 3.09 | 0.082 | 0.04 |
|  | Group simple effect: post-intervention |  |  | 26.37 | < 0.001 | 0.24 |
|  | Group × Time |  |  | 5.98 | 0.0165 | 0.07 |
| Social Distress | Latin dance group (n = 44) | 2.95 ± 0.35 | 2.75 ± 0.39 | 4.35 | 0.040 | 0.05 |
|  | Control group (n = 44) | 3.09 ± 0.53 | 3.26 ± 0.44 | 3.35 | 0.072 | 0.04 |
|  | Time main effect |  |  | 0.03 | 0.856 | 0.00 |
|  | Group main effect |  |  | 26.03 | < 0.001 | 0.23 |
|  | Group simple effect: baseline |  |  | 2.13 | 0.148 | 0.02 |
|  | Group simple effect: post-intervention |  |  | 33.14 | < 0.001 | 0.28 |
|  | Group × Time |  |  | 7.63 | 0.007 | 0.08 |
| Social Avoidance and Distress overall mean score | Latin dance group (n = 44) | 2.91 ± 0.36 | 2.72 ± 0.40 | 4.04 | 0.048 | 0.05 |
|  | Control group (n = 44) | 3.05 ± 0.47 | 3.23 ± 0.42 | 3.61 | 0.061 | 0.04 |
|  | Time main effect |  |  | 0.01 | 0.938 | 0.00 |
|  | Group main effect |  |  | 28.06 | < 0.001 | 0.25 |
|  | Group simple effect: baseline |  |  | 2.75 | 0.101 | 0.03 |
|  | Group simple effect: post-intervention |  |  | 33.43 | < 0.001 | 0.28 |
|  | Group × Time |  |  | 7.64 | 0.007 | 0.08 |

*Note.* Values are presented as M ± SD. The Bonferroni-adjusted α = 0.0167 applies only to the three social anxiety Group × Time interaction tests. The exact unrounded p value for the Social Avoidance interaction was 0.016534 and is reported as 0.0165 in the table. The simple-effects analyses were exploratory; their two-sided p values were not adjusted for multiple comparisons. ηₚ², partial eta squared.
